# Supplementary material for: A next-generation dual guide CRISPR system for genetic interaction library screening
Source: Nat Commun. 2025 Dec 6;17:561. doi: 10.1038/s41467-025-67256-9 (PMC12808759; doi:10.1038/s41467-025-67256-9)
Supplement: Supplementary file 1 — Supplementary Information [file 41467_2025_67256_MOESM1_ESM.pdf]

Supplementary Information

A next-generation dual guide CRISPR system for genetic interaction library screening

Thomas Burgold[1], Emre Karakoc[1,2], Emanuel Gonçalves[1,3,4], Inigo Barrio-Hernandez[1,2], Lisa Dwane[1], Romina Silva[1,2], Emily Souster[1,2], Mamta Sharma[1], Alexandra Beck[1,2], Gene Ching Chiek Koh[1], Lykourgos-Panagiotis Zalmas[2], Mathew J Garnett[1,2], Andrew R Bassett[1,2,#]

Supplementary figures 1-4

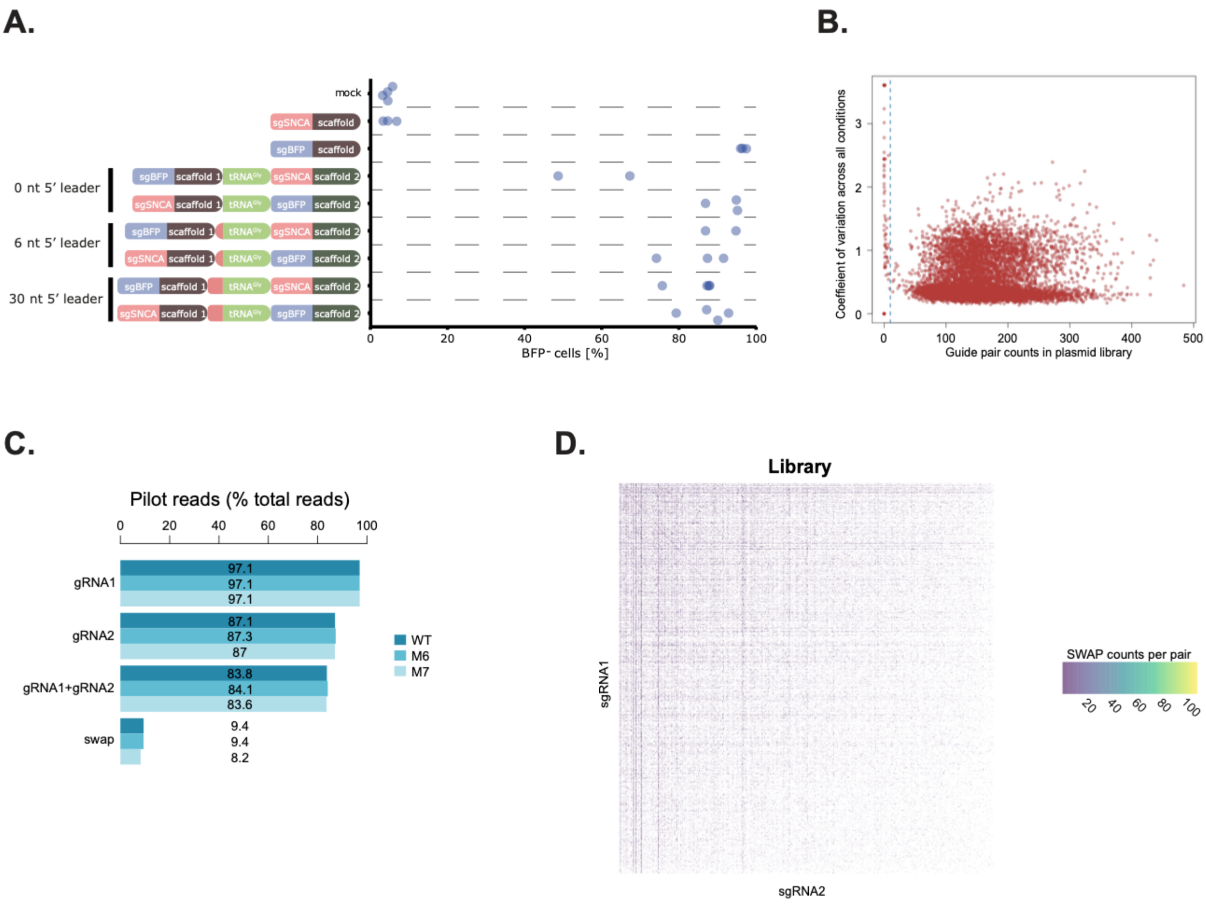

Supplementary Figure 1. Establishment of a dual guide system for pooled genetic interaction screening. (A) Editing efficiency of *BFP* (blue) guides in position 1 or 2 with different lengths of tRNA leader compared to single guide vectors. Data is shown as the percentage of BFP negative cells assessed by flow cytometry data for each biological repeat. Source data are provided as a Source Data file. (B) Distribution of raw guide pair read counts (n=8914) in pilot plasmid library (x-axis) showing coefficient of variation for each guide pair (y-axis). Read count cut-off (10 reads) is indicated by a dotted line. (C) Proportion of reads in the pilot library with perfect matches to guide RNAs in position 1 (gRNA1), position 2 (gRNA2), both positions (sgRNA1+sgRNA2) or guide pairs that were not designed in the library (Swap) split by backbone (WT, mut6, mut7). (D) Heatmap of swapped guide pairs coloured by the frequency of the pair in the cloned pilot library to assess bias in swaps. Individual guides in position 1 and 2 are shown on the x- and y-axes.

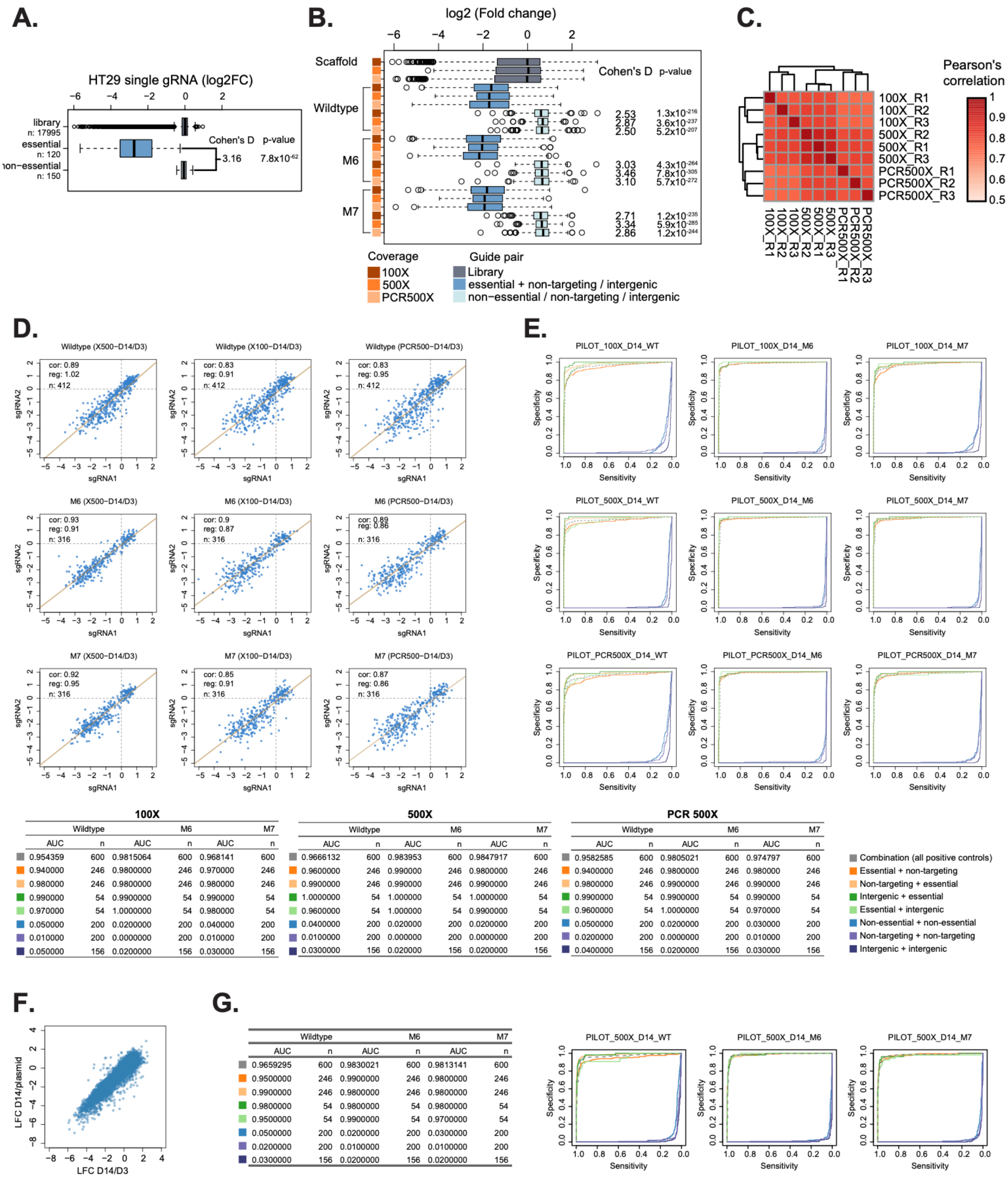

100X

	Wildtype		M6		M7	
	AUC	n	AUC	n	AUC	n
Combination (all positive controls)	0.954359	600	0.9815064	600	0.968141	600
Essential + non-targeting	0.940000	246	0.9800000	246	0.970000	246
Non-targeting + essential	0.980000	246	0.9800000	246	0.980000	246
Intergenic + essential	0.990000	54	0.9900000	54	0.990000	54
Essential + intergenic	0.970000	54	1.0000000	54	0.980000	54
Non-essential + non-essential	0.050000	200	0.0200000	200	0.040000	200
Non-targeting + non-targeting	0.010000	200	0.0000000	200	0.010000	200
Intergenic + intergenic	0.050000	156	0.0200000	156	0.030000	156

500X

	Wildtype		M6		M7	
	AUC	n	AUC	n	AUC	n
Combination (all positive controls)	0.9666132	600	0.983953	600	0.9847917	600
Essential + non-targeting	0.9600000	246	0.990000	246	0.9800000	246
Non-targeting + essential	0.9900000	246	0.990000	246	0.9900000	246
Intergenic + essential	1.0000000	54	1.000000	54	1.0000000	54
Essential + intergenic	0.9600000	54	1.000000	54	0.9900000	54
Non-essential + non-essential	0.0400000	200	0.020000	200	0.0200000	200
Non-targeting + non-targeting	0.0100000	200	0.000000	200	0.0000000	200
Intergenic + intergenic	0.0300000	156	0.020000	156	0.0200000	156

PCR 500X

	Wildtype		M6		M7	
	AUC	n	AUC	n	AUC	n
Combination (all positive controls)	0.9582585	600	0.9805021	600	0.974797	600
Essential + non-targeting	0.9400000	246	0.9800000	246	0.980000	246
Non-targeting + essential	0.9800000	246	0.9900000	246	0.990000	246
Intergenic + essential	0.9900000	54	0.9900000	54	0.990000	54
Essential + intergenic	0.9600000	54	1.0000000	54	0.970000	54
Non-essential + non-essential	0.0500000	200	0.0200000	200	0.030000	200
Non-targeting + non-targeting	0.0200000	200	0.0000000	200	0.010000	200
Intergenic + intergenic	0.0400000	156	0.0200000	156	0.030000	156

G.

	Wildtype		M6		M7	
	AUC	n	AUC	n	AUC	n
Combination (all positive controls)	0.9659295	600	0.9830021	600	0.9813141	600
Essential + non-targeting	0.9500000	246	0.9900000	246	0.9800000	246
Non-targeting + essential	0.9900000	246	0.9800000	246	0.9800000	246
Intergenic + essential	0.9800000	54	0.9800000	54	0.9800000	54
Essential + intergenic	0.9500000	54	0.9900000	54	0.9700000	54
Non-essential + non-essential	0.0500000	200	0.0200000	200	0.0300000	200
Non-targeting + non-targeting	0.0200000	200	0.0100000	200	0.0100000	200
Intergenic + intergenic	0.0300000	156	0.0200000	156	0.0200000	156

Supplementary Figure 2. Pilot genetic interaction screen identifies optimal design.
(A) Boxplot showing the log2 fold change in abundance of sgRNAs for each gene (mean of three biological repeats) between plasmid and day 14 for a published genome wide single guide screen in HT29 cells[1]. Data is grouped into guides targeting essential genes, non-essential genes as well as the entire library. Box-and-whisker plots show interquartile range (box) 1.5x range (whisker) with outliers marked, centres indicate medians. Cohen's D values and two sided t-test p-values are shown. (B) Boxplot showing the log2 fold change in abundance of sgRNAs for each gene (mean of three biological repeats per plot) between day 3 and day 14. Data is grouped into non-targeting guides or those targeting non-essential genes or intergenic regions (Non-essential / non-targeting / intergenic, n=556 per scaffold), guides targeting essential genes paired with non-targeting or intergenic guides (Essential + non-targeting / intergenic, n=600 per scaffold) or the entire pilot library (Library, n=2970). Different coverages (100x, 500x) of the screen and different backbones in position 1 (WT, M6, M7) are shown (mean of three biological replicates per coverage). Box-and-whisker plots show interquartile range (box) 1.5x range

(whisker) with outliers marked, centres indicate medians. Cohen's D and two-sided t-test p-values are shown for essential versus control genes. (C) Correlation of log2 fold change between biological repeats (R1, R2, R3) and coverages (100x, 500x or PCR500x consisting of a 100x coverage based on cells but 500x during the first round PCR) of all gene pairs. Pearson correlation coefficients are indicated on a red colour scale. (D) Correlation of the log2 fold change of sgRNA abundance with guides in position 1 (sgRNA1) and 2 (sgRNA2) separated by sgRNA scaffold in position 1 (WT, M6, M7) and coverage at which the library was screened (100x, 500x, PCR500x). Number of genes (n), pearson's correlation coefficient (cor) and gradient of regression line (reg) are indicated. (E) Recall curves of essential genes[1] depending on position within the vector and pairing with intergenic (green) or non-targeting (orange) guides. Pairs of guides targeting non-essential genes (light blue) or intergenic (purple) regions are also compared to non-targeting (dark blue) guide pairs. Graphs are shown across different sgRNA scaffolds in position 1 (WT, M6, M7) and coverage at which the library was screened (100x, 500x, PCR500x). Area under the curve (AUC) is shown in the table along with numbers of guides analysed along with number of genes (n). (F) Correlation of log2 fold change at day 14 for pilot library at 500x coverage when using day 3 (LFC D14/D3) or plasmid library (LFC D14/plasmid) as control (mean of three biological replicates, n=8914). (G) Recall curves of essential genes[1] as (E) but using plasmid library as control. Lines indicate position within vector and pairing with intergenic (green) or non-targeting (orange) guides. Pairs of guides targeting non-essential genes (light blue) or intergenic (purple) regions are also compared to non-targeting (dark blue) guide pairs. Graphs are shown across different sgRNA scaffolds in position 1 (WT, M6, M7) at 500x coverage of the library. Area under the curve (AUC) is shown in the table along with numbers of genes analysed (n).

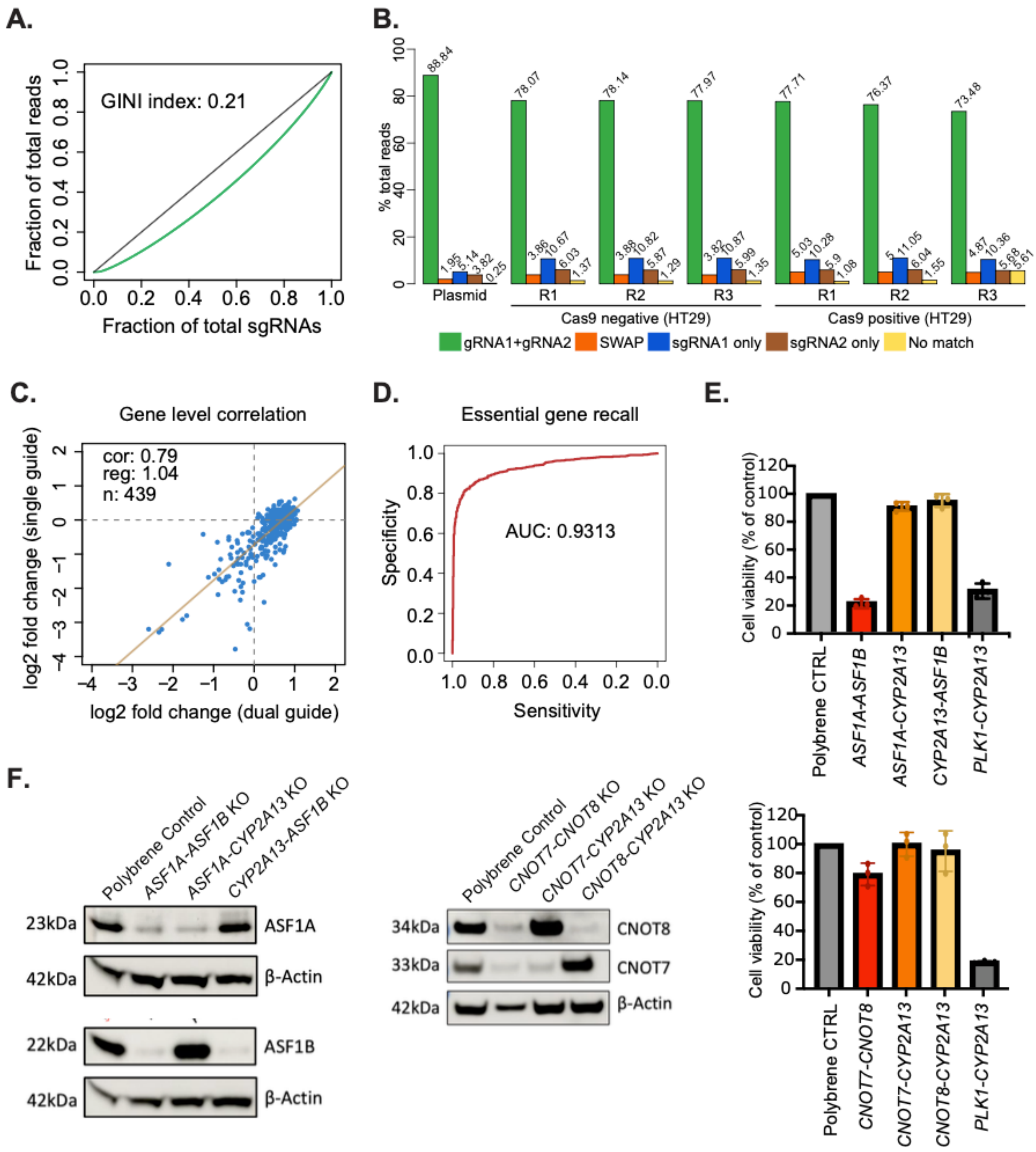

Supplementary Figure 3. Large scale genetic interaction screening identifies paralog pairs. (A) Cumulative sgRNA distribution plot to analyse skew in the cloned genetic interaction library. Gini coefficients are indicated. (B) Proportion of reads in the genetic interaction library with perfect matches to guide RNA pairs in the library (sgRNA1+sgRNA2), guide pairs that were not designed in the library (SWAP), incorrect vectors with only one guide cloned from position 1 (gRNA1_only) or 2 (gRNA2_only) or no match to any guide (No match). Values are shown for the plasmid library (Plasmid) and each biological repeat (R1, R2, R3) at day 3 post-transduction into HT-29 cells (HT-29), in either a line expressing Cas9 (Cas9 positive) or not (Cas9 negative). (C) Correlation of the log2 fold change of sgRNA abundance of 439 essential and non-essential control genes between day 3 and day 14 between dual guide vectors targeting one gene and an intergenic control and single guide vectors targeting the same genes from published data[1]. Linear regression is shown by a solid line and Pearson's correlation coefficient (cor), gradient of the regression line (reg) and number of genes (n) indicated. (D) Recall curve of essential genes for essential-intergenic or essential-non-targeting control guide pairs in screen. AUC value is indicated. (E) Endpoint cell viability measured by CellTiterGlo relative to negative control for cells targeted with dual guide vectors. Untransduced cells (Polybrene CTRL) are compared to a positive control

targeting an essential gene and a non-essential gene (*PLK1-CYP2A13*) or combinations of each paralog pair coupled with a non-essential gene (*ASF1A, ASF1B, CNOT7, CNOT8-CYP2A13*) or both paralogs (*ASF1A-ASF1B* or *CNOT7-CNOT8*). Individual data points and mean values are shown, error bars show standard deviation of three biological replicates. Source data are provided as a Source Data file. (F) Western blots for the indicated proteins in cell lysates from the samples shown in (D) excluding *PLK1-CYP2A13*.

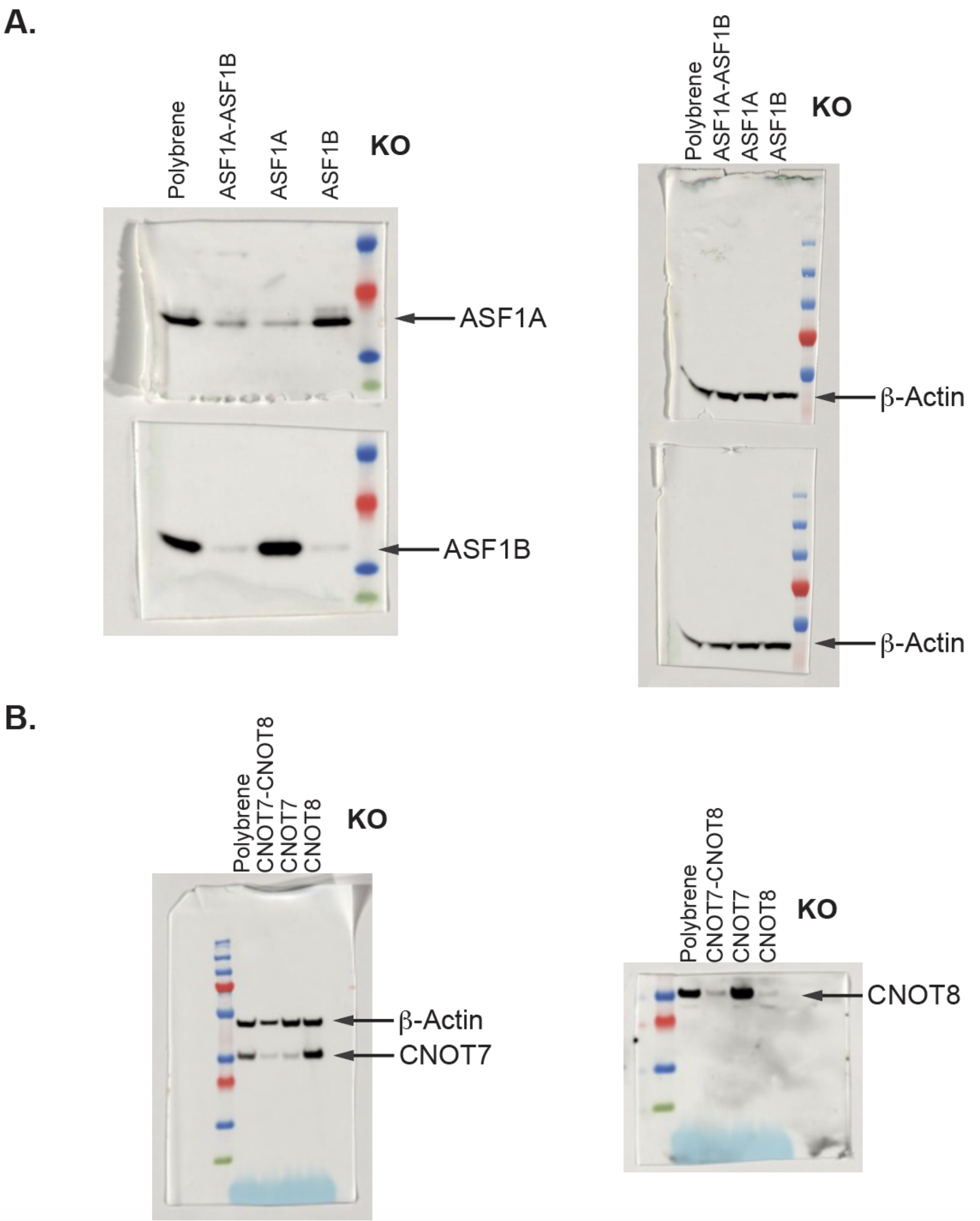

Supplementary Figure 4. Original Western blot images.
Original Western blot images for (A) ASF1A and ASF1B or (B) CNOT7 and CNOT8. Untransduced cells (Polybrene) are compared to combinations of each paralog pair coupled with a non-essential gene (ASF1A, ASF1B, CNOT7, CNOT8-CYP2A13) or both paralogs (ASF1A-ASF1B or CNOT7-CNOT8).
