# Supplementary material for: A next-generation dual guide CRISPR system for genetic interaction library screening
Source: Nat Commun. 2025 Dec 6;17:561. doi: 10.1038/s41467-025-67256-9 (PMC12808759; doi:10.1038/s41467-025-67256-9)
Supplement: Supplementary file 2 — Description of Additional Supplementary Files [file 41467_2025_67256_MOESM2_ESM.pdf]

Description of Additional Supplementary Files

File Name: Supplementary Data 1
Description: Pilot library design and results
Design of 8914 vectors for the pilot library and counts in each library. Columns show guide pair identifier (ID), category (Notes), sgRNA scaffold type (Scaffold) as well as information for each guide in the pair. This comprises WGE 45 identifier (sgRNAx_WGE_ID), target site (sgRNAx_WGE_Sequence), any library that it has been previously used in (sgRNAx_Library), gene identifier (sgRNAx_Approved_Symbol), off target information (sgRNAx_Off_Target), chromosome (sgRNAx_Chr) and start and end coordinates and strand in hg38 (sgRNA1_Start, sgRNA1_End and sgRNA1_Strand). The sequence of the first scaffold (Scaffold_Sequence), linker (Linker_Sequence), tRNA (tRNA_Sequence), sgRNA sequences (sgRNA1 or sgRNA2) and whole oligo (Oligo_Sequence) are indicated. Classification of the guide RNAs (sgRNA1_class, sgRNA2_class) and overall vector (vector_class) are shown. Raw (rawcounts) and normalised counts in the plasmid library (plasmid), of biological repeats (Rep1, Rep2, Rep3) at day 3 or day 14 post-transduction (D3, D14) and of screens performed at different coverages (100x, 500x, PCR500x) are indicated along with fold change relative to D3 (FC) and plasmid library (FC_Plasmid).
