# Supplementary material for: A next-generation dual guide CRISPR system for genetic interaction library screening
Source: Nat Commun. 2025 Dec 6;17:561. doi: 10.1038/s41467-025-67256-9 (PMC12808759; doi:10.1038/s41467-025-67256-9)
Supplement: Supplementary file 4 — Reporting Summary [file 41467_2025_67256_MOESM4_ESM.pdf]

nature portfolio

Corresponding author(s): Andrew Bassett

Last updated by author(s): 13/11/2025

Reporting Summary

Nature Portfolio wishes to improve the reproducibility of the work that we publish. This form provides structure for consistency and transparency in reporting. For further information on Nature Portfolio policies, see our Editorial Policies and the Editorial Policy Checklist.

Statistics

For all statistical analyses, confirm that the following items are present in the figure legend, table legend, main text, or Methods section.

n/a	Confirmed	
☐	☒	The exact sample size (*n*) for each experimental group/condition, given as a discrete number and unit of measurement
☐	☒	A statement on whether measurements were taken from distinct samples or whether the same sample was measured repeatedly
☐	☒	The statistical test(s) used AND whether they are one- or two-sided<br>*Only common tests should be described solely by name; describe more complex techniques in the Methods section.*
☒	☐	A description of all covariates tested
☐	☒	A description of any assumptions or corrections, such as tests of normality and adjustment for multiple comparisons
☐	☒	A full description of the statistical parameters including central tendency (e.g. means) or other basic estimates (e.g. regression coefficient) AND variation (e.g. standard deviation) or associated estimates of uncertainty (e.g. confidence intervals)
☐	☒	For null hypothesis testing, the test statistic (e.g. *F*, *t*, *r*) with confidence intervals, effect sizes, degrees of freedom and *P* value noted<br>*Give P values as exact values whenever suitable.*
☒	☐	For Bayesian analysis, information on the choice of priors and Markov chain Monte Carlo settings
☐	☒	For hierarchical and complex designs, identification of the appropriate level for tests and full reporting of outcomes
☐	☒	Estimates of effect sizes (e.g. Cohen's *d*, Pearson's *r*), indicating how they were calculated

Our web collection on statistics for biologists contains articles on many of the points above.

Software and code

Policy information about availability of computer code

Data collection	Custom code to generate libraries - see https://github.com/ibarrioh/DualGuide_COLO1/, https://github.com/EmanuelGoncalves/crispy/blob/master/notebooks/dualguide/Library2Composition.py and https://doi.org/10.5281/zenodo.17191951. for details.
Data analysis	Custom code to analyse libraries - see see https://github.com/ibarrioh/DualGuide_COLO1/ and https://doi.org/10.5281/zenodo.17191951 for details. pyCROQUET v1.6.0 (https://github.com/cancerit/pycroquet) for guide counts, Gemini v1.5.1 for genetic interaction scores (https://github.com/sellerslab/gemini).

For manuscripts utilizing custom algorithms or software that are central to the research but not yet described in published literature, software must be made available to editors and reviewers. We strongly encourage code deposition in a community repository (e.g. GitHub). See the Nature Portfolio guidelines for submitting code & software for further information.

Data

Policy information about availability of data

All manuscripts must include a data availability statement. This statement should provide the following information, where applicable:

- Accession codes, unique identifiers, or web links for publicly available datasets
- A description of any restrictions on data availability
- For clinical datasets or third party data, please ensure that the statement adheres to our policy

We have included a data availability statement describing the public access of data at Zenodo (https://doi.org/10.5281/zenodo.17191951), FigShare (https://doi.org/10.6084/m9.figshare.25533091.v1) and at the EBI-ENA (accession number ERP183979).

Research involving human participants, their data, or biological material

Policy information about studies with human participants or human data. See also policy information about sex, gender (identity/presentation), and sexual orientation and race, ethnicity and racism.

Reporting on sex and gender	No human participants or data, only one human cancer cell line.
Reporting on race, ethnicity, or other socially relevant groupings	No human participants or data, only one human cancer cell line.
Population characteristics	Not applicable
Recruitment	Not applicable
Ethics oversight	Not applicable

Note that full information on the approval of the study protocol must also be provided in the manuscript.

Field-specific reporting

Please select the one below that is the best fit for your research. If you are not sure, read the appropriate sections before making your selection.

☒ Life sciences ☐ Behavioural & social sciences ☐ Ecological, evolutionary & environmental sciences

For a reference copy of the document with all sections, see nature.com/documents/nr-reporting-summary-flat.pdf

Life sciences study design

All studies must disclose on these points even when the disclosure is negative.

Sample size	No statistical method was used to predetermine sample size, but screens were all performed in biological triplicate across at least three guide RNA pairs per target pair, which based on analysis of CRISPR screening data (Behan et al. 2019, DepMap) gave optimal performance.
Data exclusions	No data was excluded from the analysis, except for low count guides as described in the methods section.
Replication	All experiments were performed in at least biological triplicate. All attempts at replication were successful.
Randomization	Experiments were performed in biological triplicates, and controlled internally with positive and negative controls
Blinding	Blinding was not relevant, since screening experiments were pooled screens and thus controlled internally.

Reporting for specific materials, systems and methods

We require information from authors about some types of materials, experimental systems and methods used in many studies. Here, indicate whether each material, system or method listed is relevant to your study. If you are not sure if a list item applies to your research, read the appropriate section before selecting a response.

Materials & experimental systems

n/a	Involved in the study
☐	☒ Antibodies
☐	☒ Eukaryotic cell lines
☒	☐ Palaeontology and archaeology
☒	☐ Animals and other organisms
☒	☐ Clinical data
☒	☐ Dual use research of concern
☒	☐ Plants

Methods

n/a	Involved in the study
☒	☐ ChIP-seq
☐	☒ Flow cytometry
☒	☐ MRI-based neuroimaging

Antibodies

Antibodies used: β-actin (Cell Signaling 4970L, 1:1000 dilution); CNOT7 (abcam ab195587, 1:1000 dilution); CNOT8 (amsbio AMS.E-AB-62996-60, 1:1000 dilution); ASF1A (ProteinTech 22259-1-AP, 1:1000 dilution); ASF1B (ProteinTech 22258-1-AP, 1:1000 dilution); ECL Anti-rabbit IgG HRP-linked whole antibody (Amersham NA934, 1:2000 dilution).

Validation: All antibodies were validated for appropriate localisation, protein size and lack of cross reactivity with other proteins on suppliers websites. Validation of the key antibodies (other than controls) was performed by CRISPR knockout of CNOT7, CNOT8, ASF1A and ASF1B in the manuscript (supplementary figure 3 and 4) demonstrating that they were specific for the proteins in question and not cross-reactive with their orthologs.

Eukaryotic cell lines

Policy information about cell lines and Sex and Gender in Research

Cell line source(s): HT29 (female) - Wellcome Sanger Institute RRID:CVCL_0320, https://cellmodelpassports.sanger.ac.uk/passports/SIDM00136

Authentication: Whole Genome Sequencing

Mycoplasma contamination: Cell lines tested negative for mycoplasma contamination

Commonly misidentified lines (See ICLAC register): No commonly misidentified lines were used

Plants

Seed stocks: Not applicable

Novel plant genotypes: Not applicable

Authentication: Not applicable

Flow Cytometry

Plots

Confirm that:

☒ The axis labels state the marker and fluorochrome used (e.g. CD4-FITC).

☒ The axis scales are clearly visible. Include numbers along axes only for bottom left plot of group (a 'group' is an analysis of identical markers).

☒ All plots are contour plots with outliers or pseudocolor plots.

☒ A numerical value for number of cells or percentage (with statistics) is provided.

Methodology

Sample preparation: Cells were dissociated with TrypLE, quenched in media containing serum, and analysed

Instrument	Cytoflex
Software	FlowJo
Cell population abundance	Quantification of BFP positive cells was as described in the manuscript
Gating strategy	Gating was performed with negative control (cells without FP expression) and positive control (cells with FP expression) samples. Cells were gated on FSC/SSC to remove doublets. Data was confirmed by high throughput sequencing of amplicons across the edited site that gave highly comparable results.

☒ Tick this box to confirm that a figure exemplifying the gating strategy is provided in the Supplementary Information.
